# Ketone Supplementation: Meeting the Needs of the Brain in an Energy Crisis

**DOI:** 10.3389/fnut.2021.783659

**Published:** 2021-12-23

**Authors:** Angela M. Poff, Sara Moss, Maricel Soliven, Dominic P. D'Agostino

**Affiliations:** Department of Molecular Pharmacology and Physiology, Morsani College of Medicine, University of South Florida, Tampa, FL, United States

**Keywords:** ketone, ketogenic diet, exogenous ketone, energy deficit, brain metabolism

## Abstract

Diverse neurological disorders are associated with a deficit in brain energy metabolism, often characterized by acute or chronic glucose hypometabolism. Ketones serve as the brain's only significant alternative fuel and can even become the primary fuel in conditions of limited glucose availability. Thus, dietary supplementation with exogenous ketones represents a promising novel therapeutic strategy to help meet the energetic needs of the brain in an energy crisis. Preliminary evidence suggests ketosis induced by exogenous ketones may attenuate damage or improve cognitive and motor performance in neurological conditions such as seizure disorders, mild cognitive impairment, Alzheimer's disease, and neurotrauma.

## Brain Energy Metabolism

The modern human brain is uniquely large. It is over 3 times larger than in the chimpanzee, our closest living relative, as well as compared to extinct early human ancestors (1). It is by far the most energetically demanding organ, accounting for over 20% of total body energy usage while comprising only 2% of its weight (2). The fundamental processes that occur in the brain, namely neurotransmission, the electrical communication that occurs between neurons, are costly to perform and susceptible to disruption with metabolic perturbances. Thus, the brain possesses an exquisite mechanism for ensuring adequate fuel availability. Glucose serves as the primary fuel under standard conditions in the adult brain. The GLUT transporters which carry glucose into the brain, namely GLUT3, exhibit a low K_m_ and high V_max_ that helps ensure glucose uptake at a relative constant and sufficient rate independent of blood concentration (3). Ketone bodies are the primary alternative fuel for the brain with lactate serving as another important source of energy (4, 5). Under conditions of low glucose and insulin, such as during fasting, starvation, extreme exercise, or calorie or carbohydrate restriction, a portion of the fats being metabolized in the liver are shunted toward the ketogenic pathway. This pathway creates the 3 ketone bodies, collectively termed ketones: beta-hydroxybutyrate (BHB), acetoacetate (AcAc), and acetone (ACE), which can all be measured with commercially available monitoring technologies. BHB and AcAc can convert between each other via the enzyme Beta-Hydroxybutyrate Dehydrogenase (BDH1), and exist in circulation at an approximate ratio of 2:1 (BHB: AcAc), making BHB the primary ketone metabolite in the blood. AcAc can spontaneously decarboxylate to ACE which can be metabolized for energy but is rapidly exhaled by the lungs. ACE is responsible for the fruity smell of the breath that can occur when someone is in ketosis. Ketones are released into the blood and carried to extrahepatic tissues for use. Some tissues, such as the brain, heart, kidneys, and skeletal muscle, are particularly avid ketone users (6, 7). Ketone metabolism is especially important and prevalent during development and infancy. Ketones serve as key substrates for lipid biosynthesis *in utero*. In the neonate, the brain consumes up to 70% of the total energy needs (2, 8). Almost half of that energy is derived from BHB as glucose levels drop precipitously after birth, and BHB levels raise naturally to 2–3 mM (8, 9). Babies typically remain in a state of mild ketosis throughout the breastfeeding period as human breastmilk is high in medium chain triglycerides which are ketogenic fats (1, 9, 10).

Considering the substantial energy requirements of the central nervous system, it is perhaps not surprising that many neurological conditions are associated with a deficit in brain energy metabolism, either as a cause, consequence, or both. Even seemingly unrelated conditions with diverse etiologies can share this common feature, including various types of neurodegeneration, traumatic injuries, stroke, seizure disorders, and others. Neurotrauma from physical insults such as concussion or traumatic brain injury (TBI) can compromise the integrity of the neuronal cell membrane, allowing dysregulated diffusion of ions and dissipation of the membrane potential which must be repaired to restore functionality (11, 12). Reestablishing the equilibrium of ions across the cell membrane requires the use of ion pumps that are driven by ATP hydrolysis. Thus, the increased need for ATP can incur a costly energetic debt to the neurons that results in an observable energy deficit in the tissue. Simultaneously, the loss of membrane integrity allows an influx of intracellular calcium that can result in mitochondrial dysfunction that impairs oxidative metabolism and exacerbates the energy crisis. A transient increase in cerebral glucose uptake termed “hyperglycolysis” is observed within 8 days following TBI in humans but is followed by a prolonged period of glucose hypometabolism (13). This magnitude and period of metabolic depression is injury severity-dependent, can persist for days to weeks, and is associated with behavioral deficits and impaired cognitive and physical function in animals and humans (14, 15).

Glucose hypometabolism is not just a hallmark feature of physical neurological damage, however. It is also often observed in neurological and neurodegenerative diseases of varied etiology. For example, there is a notable deficit in glucose metabolism (~20–25%) in patients with Alzheimer's disease compared to healthy, age-matched controls (16). This has classically been attributed to a consequence of neuronal dysfunction and loss. However, this deficit can be observed even decades prior to the appearance of cognitive symptoms, leading some researchers to wonder if impaired glucose metabolism contributes to the pathogenesis of the disease (16). Furthermore, elevated brain tissue glucose levels and a reduced glycolytic flux are associated with severity in AD pathology in human brain samples taken postmortem (17). Similarly, impaired brain glucose metabolism is observed in numerous seizure (Sz) disorders, and has the potential to play a causative role. Heterozygous deletion of GLUT3 elicits spontaneous Szs in rats (18). Pre-clinical studies have shown a dramatic increase in glucose uptake during Sz, followed by periods of hypometabolism, which may reflect a failed attempt to meet energy needs during the Sz (19). Cerebral glucose hypometabolism is an early symptom of acquired epilepsy, a link that appears bidirectional in that hypometabolism is thought to be both a cause and consequence of Sz (20). In temporal lobe epilepsy, the most common type of refractory focal epilepsy, glucose hypometabolism is observed in the affected temporal lobe as well as several ipsilateral brain structures, whereas glucose hypermetabolism can be contralateral temporal lobe and other brain regions (21).

Thus, extensive ongoing research is investigating the degree to which deficits in energy metabolism contribute to the pathogenesis or progression of neurological disease. Novel therapeutic methods that support cerebral metabolic requirements are considered a promising avenue of research with the physiological state of ketosis being one such strategy to meet the needs of the brain in an energy crisis.

## Therapeutic Ketosis

Early work by Cahill and Owen demonstrated the degree to which the brain can rely on ketones for energy in the face of a glucose deficit (5). Obese subjects fasted for 5–6 weeks exhibited high levels of ketosis (~6 mM BHB and 1 mM AcAc). Jugular catheterization was performed to analyze arterio-venous differences in metabolites in these individuals and showed that 2/3 of cerebral fuel was being provided by BHB and AcAc. Simultaneously, urinary nitrogen excretion fell dramatically, reflecting a reduced need for muscle catabolism to provide gluconeogenic precursors in a state of starvation ketosis. To directly test the resilience of the human brain under ketotic conditions, Drenick et al. in 1972 fasted obese subjects for 2 months and then administered to them a weight-adjusted dose of insulin to induce hypoglycemia (22). Glucose levels dropped precipitously to typically pathogenic levels (~20 mg/dL), but as the subjects were in a state of therapeutic ketosis (5–7 mM BHB+AcAc), they remained asymptomatic despite the extreme hypoglycemia they experienced. This is in contrast to the usual signs and symptoms observed in the subjects when given a baseline insulin tolerance test, which included sweating, nervousness, tachycardia, hypertension, and a rise in urine catecholamine excretion. This incredible, albeit risky, experiment firmly cemented the adequacy of ketones as an alternative cerebral energy substrate, demonstrating that even in severe conditions of glucose insufficiency, ketones can protect the brain against energetic crises.

Work by Cunnane et al. has further solidified the role of ketones as not just a backup fuel, but perhaps even a *preferred* fuel for the brain, when both glucose and ketones are available. PET imaging to assess cerebral metabolic rate of ketone and glucose uptake in humans revealed that ketones are pushed into the brain at a rate directly proportional to their concentration in the blood (23). Conversely, glucose is pulled into the brain at a rate dependent on the metabolic requirement of the cell, and glucose uptake decreases inversely with an increase in ketone uptake (23). Thus, Dr. Cunnane posits that these data suggest ketones are in fact the preferred fuel for the brain, as it utilizes ketones for energy to the extent that they are available and reduces glucose metabolism accordingly. Importantly, PET imaging wherein glucose and AcAC uptake was concurrently measured in humans demonstrates that ketone uptake in the aging brain is not impaired. This is in contrast to glucose uptake which is reduced with age and to an even greater extent with mild cognitive impairment and dementia (24). Furthermore, Liquid chromatography–mass spectrometry analysis of brain metabolites following exogenous ketone ester in non-fasted mice demonstrated an increase in acetyl CoA and Kreb's Cycle intermediates which inhibited glycolysis through a negative feedback mechanism (25). Taken together, these data support the hypothesis that providing ketones to the brain could help meet energy needs regardless of the glucose status of the tissue.

In fact, *in vitro* studies have shown that ketones do directly protect neurons against hypoglycemia-induced death, an effect likely mediated by both a stimulation of mitochondrial ATP production as well as a reduction in ROS generation (26). Both *in vivo* and *in vitro* work has shown that D-BHB can substitute for glucose in hypoglycemic conditions, preserving metabolic activity, preventing oxidative stress, and attenuating neuronal death (26). This protection may also be related to ketone-induced effects on autophagy. During glucose deprivation, neurons activate autophagy as observed by an increase in LC3-II expression and number of autophagic vesicles. When treated with BHB, these neurons exhibit a rapid decline in LC3-II during glucose reintroduction, suggesting that the ketones stimulate autophagic flux which may promote neuronal survival by preventing accumulation of autophagosomes (27, 28).

There are additional proposed benefits to ketone metabolism in an energy-deficient brain. For example, compared to the 10 enzymatic steps required to convert glucose to acetyl CoA in preparation for its entry into the Kreb's cycle, BHB can be converted to acetyl CoA in just 3 reactions. Thus, ketones bypass several rate limiting enzymes such as pyruvate dehydrogenase complex which become beneficial in a state of energy deficit. Furthermore, ketones have been shown to increase the potential for ATP production due to a widening of the substrate ratio of NADH and NAD+ between Complex I and II of the mitochondrial electron transport chain (8). Thus, ketones can produce more ATP per carbon than glucose. Furthermore, a reduction in the NAD couple that occurs with ketone metabolism decreases mitochondrial ROS production. This and an increased expression of endogenous antioxidants, possibly due to metabolic and/or epigenetic effects, both result in reduced oxidative stress, another hallmark feature of neurological disorders (12). Therefore, aside from providing metabolic support to meet the energetic needs of neurons, ketones may elicit neuroprotection through distinct signaling mechanisms which are outside of the scope of this review but have been expertly described elsewhere (29).

## Exogenous Ketone Supplementation

While ketosis occurs naturally with fasting or starvation, it can also be intentionally induced by manipulation of dietary macronutrients. The ketogenic diet was developed in the early 20th Century as a method of mimicking the fasting-induced suppression of epileptic Szs, but the diet allowed for the prolonged sustainment of therapeutic ketosis. Thus, clinicians designed a diet high in fat, adequate in protein, and very low in carbohydrate to recreate the ratio of macronutrients catabolized during a fast. These conditions lower glucose and insulin, and the increase metabolism of fat induces a metabolic state of nutritional ketosis as previously described. Following the advent of antiepileptic drugs, the ketogenic diet fell out of favor until its renaissance in the 1990s. Since then, it has garnered attention once again as a proven safe and efficacious treatment for refractory (drug resistant) epilepsy. And within the last decade, significant effort has been put toward understanding what other conditions in which nutritional ketosis may provide benefit. Due to the underlying metabolic pathophysiology of many neurological disorders, these conditions have come under widespread interest and investigation. Despite this, the ketogenic diet has limitations that can complicate its clinical application. It can be viewed as restrictive by some which has the potential to reduce compliance. Additionally, side effects such as headache, GI distress, and kidney stones are possible, though typically mild and transient in nature and can be managed with proper dietary supplementation or modification of macronutrient ratios. Additionally, long term effects of chronic ketogenic diet consumption on overall and cardiovascular health are not fully understood. Encouragingly, the current state of the evidence suggests KD can be safe and feasible, and multiple biomarkers of cardiometabolic health can even improve with its use (30, 31).

Thus, in an effort to extend the practicality of ketogenic metabolic therapy, researchers have developed and tested a number of exogenous ketone supplements that release or are precursors to ketone bodies, inducing a state of physiologic ketosis regardless of dietary macronutrient intake (32, 33). Exogenous ketones (EKs) can induce a dose-dependent elevation in blood ketones and are being used in addition to standard, low carbohydrate, or ketogenic diets, depending on the needs and preferences of the individual. The major types of EKs available to date include naturally occurring ketogenic fats or synthetic compounds such as ketone salts or ketone esters. These compounds have been comprehensively reviewed elsewhere (32–34), so we will only provide a simple overview of the major types here.

Medium chain triglycerides (MCT) contain fatty acids with hydrocarbon side chains in the length of 6–12 carbons: caproic acid (C6), caprylic acid (C8), capric acid (C10), and lauric acid (C12). MCTs are found naturally in foods such as coconut oil, palm oil, and butter. MCTs can be derived from their natural sources through lipid fractionation, a process that is used to produce commercialized products MCTs that are often composed largely of caprylic (C8) and capric (C10) acid. The unique structure of MCTs allows them to be absorbed and transported through the hepatic portal vein directly to the liver where they are rapidly metabolized, partially into ketones. Thus, MCTs are considered ketogenic fats, with C8 and C10 being the most ketogenic of the group, as they elevate blood ketone levels regardless of calorie or carbohydrate content (35). MCTs have been consumed commercially for decades and appear safe (36). They can be used to induce a mild level of ketosis (typically up to 1 mM); however, the degree of ketosis induced by MCTs is limited by gastrointestinal distress, e.g. cramping and enhanced gastric motility that can occur with high doses.

Ketone salts are a synthetic form of EKs that consist of a ketone body, most often BHB, bound to a mineral ion such as Na^+^, K^+^, Ca^+2^, or Mg^+2^ (37). They are sometimes combined with MCTs in 1:1 or 1:2 mixtures to improve tolerability and prolong ketosis (32). There are numerous commercially available ketone salt products. Studies largely suggest that ketone salts can elevate blood ketones in the range of 0.5–1.5 mM for up to a few hours (37). The level of ketosis achieved by ketone salts is low to moderate due to the high mineral load and gastrointestinal intolerance associated with higher dosing. This self-limiting feature of ketone salts prevents higher therapeutic levels to be achieved, but also reduces the potential for serious side effects like ketoacidosis. Little is known regarding long-term effects of ketone salt consumption.

Ketone esters are a more potent form of synthetic EK and have the potential to truly elevate ketones to any desired level in a dose-dependent fashion. Due to this, KEs carry with them the potential for ketoacidosis if inappropriately overconsumed, and the long-term safety profile of KEs has not been fully established. Cost of synthesis and poor palatability have historically limited commercialization efforts with KEs, but recent formulations exhibit a more acceptable taste profile and price point, thus growing their popularity. A number of ketone mono- and di-esters have been developed and typically consist of ketone bodies esterified to a backbone molecule which may itself be ketogenic. The most well characterized include the (R)-3-Hydroxybutyl (R)-3-hydroxybutyrate (ketone monoester; KME) (38, 39) and the R,S-1,3-butanediol acetoacetate diester (ketone diester; KDE) (32, 40). Both KME and KDE are esterified to a ketogenic backbone molecule, 1,3-butanediol, which undergoes liver metabolism to produce BHB and can be considered an EK as a standalone agent. KME specifically produces R-BHB, the metabolically active form of BHB, while KDE produces R-BHB, S-BHB (which is metabolically inert but appears to retain signaling properties), and AcAc. KME has been shown to produce sustained ketosis (up to 4 mM) in healthy adults for 28 days when 25 mL was consumed three times daily (41). No adverse effects were observed, aside from rarely a mild nausea following consumption. KDE has not been well-tested in humans, so dosage and side effect profile is not well-established, but pre-clinical studies suggest it can induce ketosis in a dose dependent manner in animals similar to KME (32). Depending on the intended use and needs of the patient, any one of these EKs may offer the most promising or appropriate strategy.

For the many reasons outlined above, ketogenic metabolic therapy (KMT) in the form of the fasting, calorie restriction, the ketogenic diet, and most recently, EK supplementation, are promising avenues for a diverse array of neurological conditions. In the sections that follow, we will highlight data from several of such emerging areas of research that seek to investigate KMT as a means to meet the needs of the brain in an energy crisis.

## Seizure Disorders

As previously described, the ketogenic diet was developed specifically as an anticonvulsant therapy. It is remarkably successful as such, reducing Szs in refractory patients by 50% in a majority of individuals, by 90% in approximately another one quarter of patients, and completely preventing Szs in so-called “super-responders” which may apply to as many as 20% of patients (42).

The specific role of the ketone bodies in the anticonvulsant properties of ketosis is not clearly understood. Some reports show that an elevation in blood ketones is unnecessary for a low carbohydrate dietary intervention to result in Sz protection, meaning that ketone levels do not necessarily correlate with anti-convulsant effects (43, 44). However, other studies indicate the level of circulating ketone bodies does in fact correlate with the anti-convulsive effects of the dietary intervention in use (45–48), and a number of pre-clinical studies demonstrate the ability of EKs to prevent or delay Szs (34). The reason for this discrepancy may reflect varying dietary formulations, Sz phenotypes and distinct disorders studied, methods of measuring ketone bodies, and model systems used in research. Here we seek to highlight data on the reported anti-convulsant effects and mechanisms of ketones supporting the investigation of EKs for Sz disorders, though more thorough reviews of the subject have been published (34).

The anticonvulsant effect of ketones may not necessarily be related to their role as an energy metabolite. ATP-sensitive K^+^ (K_ATP_) channels are expressed in central neurons, control neuronal excitability in response to metabolic stress, and serve a protective role against Szs (49, 50). When these channels open, potassium ions (K+) move down their electrochemical gradient, lowering the action potential of the neuron. When mice are genetically modified so that their neurons and astrocytes rely on preferentially on BHB instead of glucose (mimicking the neuronal effects of ketosis) the opening of K_ATP_ channels is increased leading to reduced severity of Szs following kainate injection (51, 52). Similarly, BHB increased both stimulus evoked and basal inward-rectifying K_ATP_ channel opening in cultured mouse neurons (53). And in GAERS rats (a genetic model of absence epilepsy), rapid firing occurs in GABAergic substantia nigra pars reticulata (SNr) neurons at the onset of Sz but decrease back to basal levels upon its cessation (54). BHB and AcAc decreased firing rates in cultured SNr neurons (49), an effect that was not seen in the absence of K_ATP_ channel opening, also pointing to this potential mechanistic role.

GABA appears to be another key player in the anticonvulsant properties of ketosis. GABA has been shown to decrease in various brain regions prior to the onset of Sz (55). Sz susceptible Mongolian gerbils exhibit lower densities of GABA-benzodiazepine receptors in the SN, and the use of GABA promoting compounds has shown to be protective against Sz activity in many model systems (55). The decrease in firing rate seen in cultured GABAergic SNr neurons after exposure to ketone bodies is normalized when exposed to a GABA_B_ receptor blocker, indicating that GABA_b_ signaling is important in this mechanism (49). Thujone, another chemical convulsant agent, is a GABA_A_ receptor antagonist whose Szs can be attenuated with AcAc, implicating an increase in GABA_A_ signaling as a therapeutic mechanism (56). Furthermore, AcAc and BHB can increase GABA synaptosomal concentration and formation rate in rat neurons (57). In a mouse model of Angelman syndrome, KDE attenuated audiogenic and kainic acid-induced seizures (58). This effect was also associated with an increase in hippocampal GAD65 and GAD67 expression, enzymes which convert glutamate to GABA, and an increase in GABA/glutamate ratio in KDE-treated mice.

Additionally, the effect of ketone bodies on vesicular glutamate transporters (VGLUTs) may confer anticonvulsant properties in some settings (59). VGLUTs are activated by Cl^−^ binding leading to a release of the excitatory neurotransmitter glutamate which occurs during glutamate excitotoxicity that contribute to Sz activity. Ketone bodies compete with Cl- to inactivate VGLUTs and can therefore suppress neuronal excitability by reducing presynaptic glutamate release (59). Exposure to a combination of BHB and AcAc has been effective in protecting rat neocortical neurons against glutamate excitotoxicity (60). In this study ketone bodies reduced neuronal death, altered membrane properties, and reduced mitochondrial production of reactive oxygen species (ROS) and their excitotoxic activities. Importantly, the ketones increased mitochondrial NADH oxidation but did not affect the levels of glutathione (an antioxidant). These results indicate ketone bodies can increase the NAD+/NADH ratio which can reduce free radical formation due to glutamate. Numerous studies have demonstrated that ketones elicit antioxidant effects, such as one wherein exposure to a combination of BHB and AcAc has protected against hydrogen peroxide toxicity, reducing ROS and mitigating impairment of long-term potentiation in rat neocortical and hippocampal neurons (61, 62).

AcAc has been shown to elicit anticonvulsant properties since 1935 when Keith and Stavraky used it to protect rabbits from thujone induced Szs (63). Since then, AcAc has been effective in protecting Frings audiogenic Sz-susceptible mice from sensory induced tonic Szs, as well as rats from 4-aminopyridine induced tonic Szs (59, 64). ACE is also effective in protecting Frings mice from sensory induced tonic Szs, as well as protecting rats from electroshock induced tonic-clonic Szs, pentylenetetrazole (PTZ)- induced absence Szs, and AY-9944 induced chronic absence Szs (64, 65). Additionally, ACE is effective in protecting mice from PTZ-induced clonic Szs as well as from 4-aminopyridine induced tonic Szs (66). The anti-convulsant properties of intraperitoneal administration of ACE appears attributable to ACE itself, and not the downstream metabolites (66).

BHB does not seem to elicit as potent anticonvulsant properties as AcAc or ACE, but may retain some efficacy as such (34). A combination treatment of BHB and KDE has been shown to mitigate the absence Sz phenotype of Wistar Albino Glaxo/Rijswijk (WAG/Rij) rats (67). And KDE and KDE+MCT combination treatments were effective in preventing hyperbaric oxygen induced tonic-clonic Szs in rats, but BHB in ketone salt form, and 1,3-butanediol which metabolizes to BHB, were not (40, 68). BHB was also not effective in protecting Frings audiogenic Sz-susceptible mice from sensory induced tonic Szs (64). Interestingly, a number of pre-clinical studies have suggested that specific MCTs such as C10:0 capric acid, may elicit anti-Sz properties through mechanisms independent of ketone metabolism and signaling (69, 70). For example, capric acid increased Sz threshold in the 6Hz psychomotor and maximal electroshock test Sz models (MEST); however, it did not affect PTZ-induced Szs (71). Interestingly, caprylic acid (C8:0 MCFA) increased Sz threshold in the 6Hz psychomotor and i.v. PTZ-induced Sz models, but not in the MEST model (72).

## Mild Cognitive Impairment And Alzheimer's Disease

Emerging applications for EKs also include age-related and pathological cognitive impairment. Mild cognitive impairment (MCI) is an early stage memory or cognitive ability loss in individuals who retain independent daily living activities that often precedes development of dementia such as Alzheimer's disease (73). As previously mentioned, glucose but not ketone metabolism is impaired in MCI. In a sepsis surviving mouse model of MCI, subcutaneous administration of BHB improved behaviors of learning and memory (74). And recently, in a randomized controlled trial, 6 months of consuming a MCT-based ketogenic drink improved recall, verbal fluency, confrontation naming, and visual attention and task switching, symptoms in humans with MCI (75). Some of the observed benefits correlated positively with blood ketone concentration, supporting a potential central role.

Most age-related neurodegenerative diseases are characterized by cognitive impairment, with one of the most common being Alzheimer's disease (AD). KMT is considered a promising therapeutic strategy for this devastating disorder. The pathology of AD is not fully understood however there is consensus that the key features of the disease include amyloid beta plaques, tau protein (found in neurofibrillary tangles), and compromised glucose metabolism as previously described. The pre-clinical stages of Alzheimer's disease embody a prolonged period of glucose hypometabolism and accumulating damage from Aβ plaques and tau tangles. Astrocytes house the glutamine-glutamate neurotransmitter cycle, and over time the damage results in oxidative stress, reduced noradrenergic input, and diminished glial glutamic activity that disrupts synaptic function. However, during this pre-clinical stage and even into full manifestation, AD brains appear to maintain a consistent ability to metabolize ketone bodies (16). As a result, some have suggested that KMT for AD may only require a modest elevation in ketones while still rendering significant benefits. This leads to postulation that early KMT could likely render even greater efficacy (76). Studies support the hypothesis that by supplying an alternative fuel to the brain, KMT can preserve neuronal function (24). One of the primary effects of glucose hypometabolism in AD is the disruption of the TCA (tricarboxylic acid) cycle which feeds into the activity of neurotransmitters, a phenomenon that KMT helps to restore (77). But the potential role of ketosis in targeting AD goes beyond the energy deficit, and KMT may also reduce neuronal hyperexcitability caused by prolonged glucose hypometabolism and reduce the neuroinflammation caused by tau and Aβ, offering a multi-pronged treatment approach.

In a cellular study, rat hippocampal cells were treated with Aβ, the ketone BHB, or a combination of the two. Of the three, death rate was highest in the cell group treated exclusively with Aβ. Whereas, in the group that received both Aβ and BHB, the number of surviving cells was almost doubled. In fact, BHB was found to act as a sort of growth factor that attenuated the effects of Aβ toxicity (78). Although mitigation of glucose hypometabolism will inherently reduce some inflammation, studies show KMT may elicit such effects in AD through other mechanisms. For example, exogenous BHB reduced neuroinflammation via inhibition of the NLRP3 inflammasome in an AD mouse model (5XFAD), an effect that correlated with an observed decrease in microgliosis. The mice that were treated with BHB were also found to have fewer Aβ plaques (79). The protective properties of BHB in the context of AD are further demonstrated in its ability to weaken the inflammatory reactions facilitated by Human apolipoprotein-E (ApoE), which has long been strongly associated with neuroinflammatory pathology of AD along with many other diseases (80). Furthermore, the cognitive decline seen in AD patient is largely linked to disrupted synaptic activity. It was found that KME treatment rendered higher hippocampal concentrations of citrate and α-ketoglutarate which are precursors to neurotransmitters aspartate and glutamate, revealing another mechanism through which KMT may help mitigate the decreased glial glutamic activity in AD pathology. In the same study, KME-treated mice displayed more exploratory behavior and increased N-acetyl-aspartate, which is a biomarker used in AD diagnosis and signifies neuronal viability (77).

The aforementioned pre-clinical findings would not be so significant if they lacked the ability to translate into humans, but encouraging early-stage clinical findings are providing great hope for the victims and caregivers of this devastating disease. In a 2015 case report, KMT in the form of MCT and KME was administered to an APOE4 positive male with early onset, sporadic AD (81). The patient was experiencing rapid progression of disease with severe memory loss and an inability to perform several activities of daily living. MRI revealed diffuse involutional changes in the frontal and parietal lobes with atrophy of the amygdala and hippocamus, and his Mini-Mental State Examination (MMSE) score declined from 23 to 12 in the 4 years prior to KMT. The patient initiated KMT in the form of MCT oil and coconut oil, sources of medium chain fatty acids. Within 3 months of KMT, the patient experienced remarkable cognitive and physical improvements and his MMSE score improved from 12 to 20. Additionally, his Alzheimer's Disease Assessment Scale-Cognitive (ADAS-Cog) improved by 6 points and his Activities of Daily Living (ADLs) score improved by 14 points over that same time period. No changes in brain atrophy were observed via MRI from June 2008 to April 2010, suggesting stabilization of disease. The patient reportedly improved in many aspects of daily living, regaining the ability to recite and write the alphabet, and dress himself which he had previously lost. He also demonstrated improvements in abstract thinking, insight, and sense of humor. The patient himself reported feeling happier and more energetic, and eventually experienced improvements in memory retrieval and regained the ability to perform more complex tasks of daily living. Over time, his caregiver fine-tuned his dose and regimen of administration, eventually settling on consuming MCT and KME 3-4x daily. Importantly, there were no adverse effects observed in the patient over this two-year study. Limited interpretation can be drawn from a case report such as this, but the reported benefits are encouraging and supportive of further clinical investigation.

Additionally, an oral ketogenic compound and prescription medical food called AC-1202 (tradename Axona) was developed by Accera, Inc. as an AD therapy. AC-1202 elevates blood ketone levels as it contains MCFAs which are natural ketogenic precursors. AC-1202 was evaluated in a randomized, double-blind, placebo-controlled, multicenter trial in patients with mild to moderate AD (82). AC-1202 induced a mild level of ketosis in patients (up to 0.3–0.4 mM), which was significantly higher than placebo controls. AC-1202 treatment induced small improvements in ADAS-Cog, MMSE, and ADCS-CGIC (AD Cooperative Study—Clinical Global Impression of Change) scores compared to placebo in some subgroups of AD patients tested. The modest improvements observed in this study may potentially be due to the comparatively low level of ketosis induced by AC-1202, considering what is attainable with KME or similar EKs. More recently, another small trial evaluating Axona in 22 Japanese patients with mild-to-moderate sporadic AD reported enhanced memory function with no difference between APOE4 positive or negative patients (83). Success was also reported in another Japanese trial where subjects with mild-to-moderate AD were administered an MCT-containing ketogenic formula called Ketonformula for 12 weeks (84). The patients exhibited improvements in verbal memory and processing speed over the course of the study. And in 2020, a group of elderly adults at risk for Alzheimer's were put on either a Mediterranean ketogenic diet (MMKD) or the American Heart Association diet (AHAD). Only those that received the MMKD showed a positive shift in CSF biomarkers of Aβ and tau, indicative of less aggregating of these proteins in the brain. The MMKD group was also associated with increased cerebral perfusion and exhibited a dietary compliance rate of 90%, suggesting that this dietary regimen is feasible in the patient population (85).

Therapeutic benefit may depend on the genetic background of the affected individual. In a double-blind randomized placebo-controlled study of 53 mild to moderate AD patients, the Chinese version of the cognitive subscale of the Alzheimer's Diseases Assessment was used to compare cognitive improvement between a group treated with MCTs and placebo controls. After 90 days of treatment and monitoring, there was no significance difference in performance between the two groups, except for ApoE -/- patients which showed improvements compared to the placebo patients (86). This is similar to the Axona trial mentioned above, in which for many of the parameters tested, AC-1202 did not affect performance of ApoE4 positive patients on tests (82). There are currently a number of registered clinical trials investigating exogenous ketones in the form of medium chain triglycerides, ketone salts, or ketone esters in MCI or AD (e.g., ClinicalTrials.gov Identifiers NCT04466735, NCT02551419, NCT00777010, NCT02984540, NCT03472664, NCT04817176, NCT02521818, NCT03130036, NCT03319173, NCT04968041).

## Neurotrauma

### Traumatic Brain Injury

Various types of neurotrauma represent another promising field for KMT (87). As previously described, physical damage to the nervous tissue induces critical energy imbalances that must be repaired to regain function. Thus, supplying an alternative fuel to the nervous system in such a setting may attenuate damage. A number of reports have investigated the use of KMT for TBI, often in the form of KD but some exploring EKs as well. In one early study, ^14^C-labeled BHB infusion following injury in the controlled cortical impact (CCI) rat model revealed enhanced cerebral ketone uptake, CO_2_ production, and ATP concentration (88). In a fluid percussion injury model of repetitive mild TBI, adolescent rats (postnatal day 35; PND35) fed a KD showed improved motor performance, an increase in cortical N-acetylaspartate:creatine staining, and a decrease in cortical microglial beclin-1 expression, suggesting potential prevention of neurodegeneration (89). In a *Drosophila* model of high-impact trauma, a BHB supplemented KD attenuated post-injury aggression (90). This appeared to be mediated by BHB effects on ATP-sensitive potassium channels, a known signaling mechanism of ketones, as pharmacological inhibition of these channels abolished, and activation mimicked, the effects of BHB in this model. In another study on adolescent rats, animals were fed a KD either pre-or post-injury using the lateral impact technique, a model of mild TBI (91). Timing of intervention influenced systemic and molecular results. Rats pre-treated with KD demonstrated a reduction in balance and motor impairments and an increase in exploratory behavior while post-injury KD treatment reduced anxiety and depressive-like behaviors. Genes of interest such as Fgf2, Iba1, Opa1, and Sirt1 were also differentially influenced by brain region and timing of KD treatment.

Age of subject may influence outcomes as well. In a rat CCI model, PND35 and PND45, but not PND17 and PND65, rats exhibited a reduction in contusion volume with KD therapy (92). Similarly, a study looking at KD treatment on TBI outcome in young (PND35) vs. adult (PND70) rats found that ketone uptake and ATP, creatine, and phosphocreatine levels were increased in the young but not adult rats (93). The authors of these papers suggest that younger brains may be more amenable to KMT following TBI due to an inherent greater capacity to adapt to ketone metabolism. Currently, there are four registered clinical trials evaluating ketogenic therapy for TBI (ClinicalTrials.gov, Identifiers: NCT04933448, NCT04530032, NCT03982602, NCT04308577).

### Spinal Cord Injury

SCI is another common form of neurotrauma that may be susceptible to treatment with KMT. In a rat model of acute cervical SCI, animals fed a KD following C5 hemi-contusion injury exhibited increased forepaw usage and range of motion, enhanced recovery of wrist and digit movement, and improved pellet retrieval capability (94). The benefits endured following a return to standard diet after 3 months of treatment. KD rats also exhibited smaller lesions and gray matter sparing compared to the control animals. Blockade of the monocarboxylate transporters that transport ketones into the tissues was sufficient to prevent the benefits of KD therapy, suggesting the neuroprotective effects were mediated by the ketones themselves. Similarly, rats treated with exogenous BHB in a moderate-severe thoracic (T9-T10) spinal contusion injury reduced motor neuron loss, microglial activation, oxidative stress, and NLRP3 inflammasome activation while improving mitochondrial function (95). Mechanisms involving inflammation and oxidative stress are a common thread in these reports. In another pre-clinical study, rats treated with intrathecal injection of BHB and exposed to a C5 hemi-contusion SCI experienced improved behavioral and electrophysiological recovery along with a suppression of microglial and NLRP3 inflammasome activation (96). Accompanying *in vitro* studies revealed similar findings, with BHB inhibiting the LPS+ATP inflammatory response in BV2 cells by reducing NLRP3 expression. The same group reported that ketosis induced by exogenous BHB, every other day fasting, and ketogenic diet reduced ROS expression and oxidative stress in this model by suppression of Class I HDACs (97, 98). The therapeutic effects may also be linked to NF-κB signaling, as rats fed a ketogenic diet following a C7 hemi-contusion exhibited improved functional recovery that was associated with a reduction in NF-κB, TNF-α, IL-1β, IFN-γ, oxidative stress, and an activation of NRF2 (99). There is currently one registered clinical trial evaluating KMT in the form of the ketogenic diet in spinal cord injury patients (ClinicalTrials.gov Identifier NCT03509571).

### Stroke

Stroke is yet another condition for which KMT is heralded as a promising therapy and has been discussed for well over a decade. In 2012, a systematic review of the pre-clinical data was published that identified 16 studies meeting predefined criteria and concluded that there is objective evidence suggesting ketosis elicits beneficial effects in experimental models of stroke (100). The studies included data from a total of 773 animal subjects, and approximately 20% utilized KD, 30% utilized EKs, and 50% utilized calorie restriction to induce a ketogenic state. Most also included pre-treated animals with KMT prior to stroke induction. Though different strains of animal, stroke models, methods of KMT induction, and outcome measures were used, the systematic review determined there was an overall significant protective effect of ketosis on outcome following cerebral ischemia, greatly supporting continued investigation. More recent work has continued to report encouraging data and has begun to uncover mechanisms of action (101–104). Rats fed a MCT-oil supplemented ketogenic diet beginning 3 days prior to an induced ischemic stroke of the middle cerebral artery (MCA) exhibited improvements in motor function compared to control animals, including the ability to move faster and adjust steps more efficiently on a beam, and to use their forepaws more symmetrically in a transparent cylinder (105). Similarly, rats treated with exogenous BHB 1 h following a cortical ischemic injury exhibited less oxidative stress, astrogliosis, and neuronal death than controls along with improved neuronal functioning and sensorimotor performance in the withdrawal reflex test (102).

In another study, rats were fed KD for 3 weeks prior to MCA occlusion/reperfusion injury and as an *in vitro* companion, SH-SY-5Y cells were pre-treated with BHB then exposed to oxygen-glucose deprivation/reoxygenation (103). In both settings, data suggested that ketosis prevented ER stress and protected mitochondrial integrity by reducing NLRP3 inflammasome activation, with the KD-treated animals also exhibiting a reduction in infarct size, decrease in apoptotic cells, and improvement in neurological score. Alternatively, some data suggests that adenosine and HIF-1 signaling may play an important role. Three weeks of preconditioning with KD reduced infarct volume and increased regional cerebral blood flow in rats given reversible MCA occlusion (104). Extracellular adenosine was also elevated by ketosis, in both the ischemic and reperfusion phases of injury, and was associated with an upregulation of HIF-1 pathway activation. Signaling through the adenosine A1 receptor was required for these beneficial effects, as its blockade abolished the therapeutic effects both *in vivo* and *in vitro*. There are a handful of registered clinical trials evaluating versions of ketogenic diets as a therapeutic for stroke (ClinicalTrials.gov Identifiers: NCT01820663, NCT01997749, NCT04184076, NCT00639730).

## Conclusion

Encouraging preliminary evidence suggests that EKs could potentially provide significant protection against conditions associated with brain energy deficit, including seizure disorders, neurodegeneration, and traumatic injuries to the nervous system. If proven effective, EKs could be easily and rapidly introduced as a more feasible option to induce therapeutic ketosis than classic methods of dietary manipulation such as the KD. Ketogenic therapies hold the added benefit of ease of monitoring. Ketones can be readily measured in the urine, blood, and breath with commercially available point of care meters while real-time, continuous ketone monitoring systems that are now in development may become available soon (106). Additionally, EKs could be used in conjunction with KD therapy to more rapidly induce a higher level of ketones, especially in settings where the temporal window of opportunity to treat may be limited, or in situations where contraindications or personal preferences undermine the ability to use KD therapy.

## Author Contributions

AP, SM, MS, and DD'A contributed to the conceptual design and writing of the manuscript. All authors contributed to manuscript revision, read, and approved the submitted version.

## Conflict of Interest

At the time of this publication: DD'A and AP are inventors on the following patents: DD'A, AP, and Patrick Arnold: “Targeting Cancer with Metabolic Therapy and Hyperbaric Oxygen” (Patent Number: 9801903). DD'A is the owner of Ketone Technologies LLC, which does consulting and public speaking events. AP is a scientific advisor to Pruvit Ventures and owner of Poff Medical Consulting and Communications, LLC and Metabolic Health Initiative, LLC. DD'A and AP are inventors on pending patent “Compositions and Methods for Weight Loss Maintenance.” DD'A is an inventor on pending patent “Prevention of Muscle Wasting with Ketone Supplementation.” At the time of this publication, pending patents were still under review. Should patents become accepted and royalties ever accrue, AP and DD'A will receive a share under the patent terms prescribed by the University of South Florida. The remaining authors declare that the research was conducted in the absence of any commercial or financial relationships that could be construed as a potential conflict of interest.

## Publisher's Note

All claims expressed in this article are solely those of the authors and do not necessarily represent those of their affiliated organizations, or those of the publisher, the editors and the reviewers. Any product that may be evaluated in this article, or claim that may be made by its manufacturer, is not guaranteed or endorsed by the publisher.
